# Rare genetic variation in UNC13A may modify survival in amyotrophic lateral sclerosis

**DOI:** 10.1080/21678421.2016.1213852

**Published:** 2016-09-01

**Authors:** Benjamin Gaastra, Aleksey Shatunov, Sara Pulit, Ashley R. Jones, William Sproviero, Alexandra Gillett, Zhongbo Chen, Janine Kirby, Isabella Fogh, John F. Powell, P. Nigel Leigh, Karen E. Morrison, Pamela J. Shaw, Christopher E. Shaw, Leonard H. van den Berg, Jan H. Veldink, Cathryn M. Lewis, Ammar Al-Chalabi

**Affiliations:** ^a^ Maurice Wohl Clinical Neuroscience Institute, King’s College London, Institute of Psychiatry, Psychology and Neuroscience, London, UK; ^b^ University Medical Centre Utrecht, Utrecht, The Netherlands; ^c^ Department of Statistical Genetics, King’s College London, Institute of Psychiatry, Psychology and Neuroscience, London, UK; ^d^ Sheffield Institute for Translational Neuroscience, Sheffield, UK; ^e^ Brighton and Sussex Medical School, Brighton, UK; ^f^ Faculty of Medicine, University of Southampton, SouthamptonUK

**Keywords:** ALS, survival, UNC13A, genetic modifiers, sequencing

## Abstract

Our objective was to identify whether rare genetic variation in amyotrophic lateral sclerosis (ALS) candidate survival genes modifies ALS survival. Candidate genes were selected based on evidence for modifying ALS survival. Each tail of the extreme 1.5% of survival was selected from the UK MND DNA Bank and all samples available underwent whole genome sequencing. A replication set from the Netherlands was used for validation. Sequences of candidate survival genes were extracted and variants passing quality control with a minor allele frequency ≤0.05 were selected for association testing. Analysis was by burden testing using SKAT.

Candidate survival genes *UNC13A*, *KIFAP3*, and *EPHA4* were tested for association in a UK sample comprising 25 short survivors and 25 long survivors. Results showed that only SNVs in *UNC13A* were associated with survival (*p* = 6.57 × 10^−3^). SNV rs10419420:G > A was found exclusively in long survivors (3/25) and rs4808092:G > A exclusively in short survivors (4/25). These findings were not replicated in a Dutch sample. In conclusion, population specific rare variants of *UNC13A* may modulate survival in ALS.

## Introduction

Amyotrophic lateral sclerosis (ALS) is a neurodegenerative disease predominantly of motor neurons, resulting in progressive weakness of voluntary muscles and death from respiratory failure due to diaphragmatic paralysis, typically within three years of onset.

There is a strong genetic contribution to ALS risk. Approximately 5% to 20% of cases have a family history of ALS or frontotemporal dementia; the remaining cases are sporadic, having no known family history. Even in apparently sporadic cases, twin and population studies estimate the heritability to be about 60% (2). Genes for ALS have now been identified in about 70% of those with a family history and 15% of those without. Surprisingly, risk genes do not strongly predict phenotype in most cases, with only a few having any reliable relationship with prognosis. For example, homozygosity for the p.D91A mutation of *SOD1* is associated with slowly progressive ALS, and the p.A5V mutation of *SOD1* with rapid disease, but such a relationship is unusual.

Despite the very poor prognosis, there is considerable variation in survival rate: up to 10% of people with ALS live more than eight years from first symptoms (3). A number of common gene variants associated with ALS survival have been identified through genome-wide association studies and animal models. These include variants in *UNC13A*, *EPHA4*, and *KIFAP3* (4–7).

The *UNC13A* association with survival has been replicated, but the *KIFAP3* finding has failed to replicate in follow-up analyses (8,9). The association of *EPHA4* variants with survival used a combination of functional and genetic approaches but has not been found in genetic association studies alone. Variants that confer robust associations to survival may offer important future therapeutic targets because patients present after symptom onset and ALS is rare, making primary prevention unfeasible (10). Survival genes could potentially be targeted directly, or their product augmented to improve ALS survival. Genetic variation in survival genes could also be used to predict prognosis and to help forecast response to future therapeutic interventions, both of which would be valuable in counselling patients and for clinical trial design (11).

It remains to be seen if large-scale sequencing studies identify rare variants in additional genes which might also modify survival. Rare genetic variation is either rare because it is recent in evolutionary terms or because it generates a deleterious phenotype and is therefore selected against. As a result, harmful rare variants might be enriched in a phenotypically extreme sample (12) and may have gone undetected in genetic association studies to date, which have focused primarily on common variation. We therefore searched for rare genetic variation in candidate survival genes in individuals showing extreme survival phenotypes in ALS, with either very short survival or very long survival.

## Materials and methods

### Candidate survival gene selection

Survival genes of interest were identified by literature review. MEDLINE and EMBASE were searched in June 2015 using the key words ‘amyotrophic lateral sclerosis’ OR ‘ALS’ OR ‘motor neuron disease’ OR ‘MND’ AND ‘survival’ AND ‘gene’. Further papers were identified by discussion with known research groups. Any gene was included if variants in or near the gene were identified as associated with ALS survival in whole-genome analysis. Studies analysing candidate genes or a restricted subset of the genome were excluded.

### Ethics

Informed consent was obtained from all subjects included in the study. The study was approved by the Trent Research Ethics Committee 08/H0405/60 and by the Medical Ethics Review Board at the University Medical Centre Utrecht 05_067/E. Patients were identified from the UK National DNA Bank for Motor Neuron Disease Research (MND DNA bank) and King’s MND DNA bank. Survival was measured from symptom onset and information collected by follow-up of patients until death or last clinic visit. Informed written consent was obtained from all participants for participation in genetic research.

### Samples

The top and bottom 1.5% of ALS patients by survival were identified (25 patients from each tail of the distribution). All patients were classified as definite or probable ALS according to the El Escorial criteria and had no family history of ALS. Sample ancestry and relatedness were evaluated by principal components analysis and relationship matrices.

### Procedures

Whole-genome sequencing was performed by Illumina (San Diego, CA, USA) using the HiSeq2000 platform. All sample data were processed with the Illumina Whole Human Genome Sequencing Informatics Pipeline. High-quality sequence reads were aligned using the iSAAC Sequence Aligner And Counter and variant calling was performed using the iSAAC Variant Caller. Quality control measures were applied to single nucleotide variation (SNV) data. SNVs were excluded from analysis using the following quality control parameters: if the locus had heterozygous genotype in a haploid region, read depth was greater than 3x the mean chromosome read depth; the fraction of base calls filtered out at a site was >0.3 SNV contextual homopolymer; length exceeded 6; SNV strand bias value exceeded 10; or genotype quality assuming variant position was <30.

### Replication studies

For replication, a second set of samples from the Netherlands was studied. Samples were whole-genome sequenced on the Illumina HiSeq 2000 platform and underwent alignment and variant calling identical to that performed in the UK samples. Samples were cleaned based on depth of coverage, Ti/Tv ratio, missingness, relatedness, ancestry evaluated by principal component analysis, a gender check (to identify mismatches), inbreeding, and genotyping-sequence concordance (made possible by genotyping data generated on the Omni 2.5M for all samples). After QC, short-term survivors (*n* = 368) who lived at most two years from symptom onset and long-term survivors (*n* = 91) who lived at least six years after symptom onset were selected for association testing.

### Statistical analysis

For discovery, patients were dichotomised into short survival and long survival groups, and analysed by group. For genes meeting criteria for inclusion as candidate survival genes, genotype data on variants lying within genes was extracted, including introns and 500bp upstream and downstream. A gene-based SKAT test (13) was conducted to compare burden of rare variation in the two groups. SKAT analysis was conducted in Variant Tools (14) and Variant Association Tools (15) by implementation of the R package ‘SKAT’ (http://cran.r-project.org/web/packages/SKAT/). This implementation of SKAT generates empirical p-values and uses methods for small sample size adjustment (13). Variants with minor allele frequency (MAF) ≤ 0.05 in this dataset were included in the SKAT burden analysis in keeping with previous extreme phenotype studies (16). Bonferroni correction for the number of genes tested was used to account for multiple testing.

To allow covariate analysis with the common variant rs12608932:A > C in *UNC13A* previously associated with ALS and with ALS survival, this variant was genotyped using Illumina 2.5M beadchip microarrays, performed at the same time as sequencing.

Identification of rare individual SNVs driving the SKAT analysis result was conducted with PLINK/SEQ software version 0.1 (https://atgu.mgh.harvard.edu/plinkseq). Haploview was used to analyse linkage disequilibrium between SNPs (17). SNAP (https://www.broadinstitute.org/mpg/snap/) was used to identify long-range linkage disequilibrium.

To estimate population frequencies of variants, we used the 503 European ancestry samples from the 1000 Genomes dataset.

## Results

Literature review identified 761 papers referring to ALS survival genes. Three genes passed our inclusion criteria: *UNC13A*, identified from genome-wide association studies; *EPHA4*, identified by a genome-wide morpholino-based zebrafish knockdown screen followed by testing in humans; and *KIFAP3*, identified in a genome-wide association study, although not subsequently confirmed as a survival gene in other studies (5–9).

**Figure 1.**
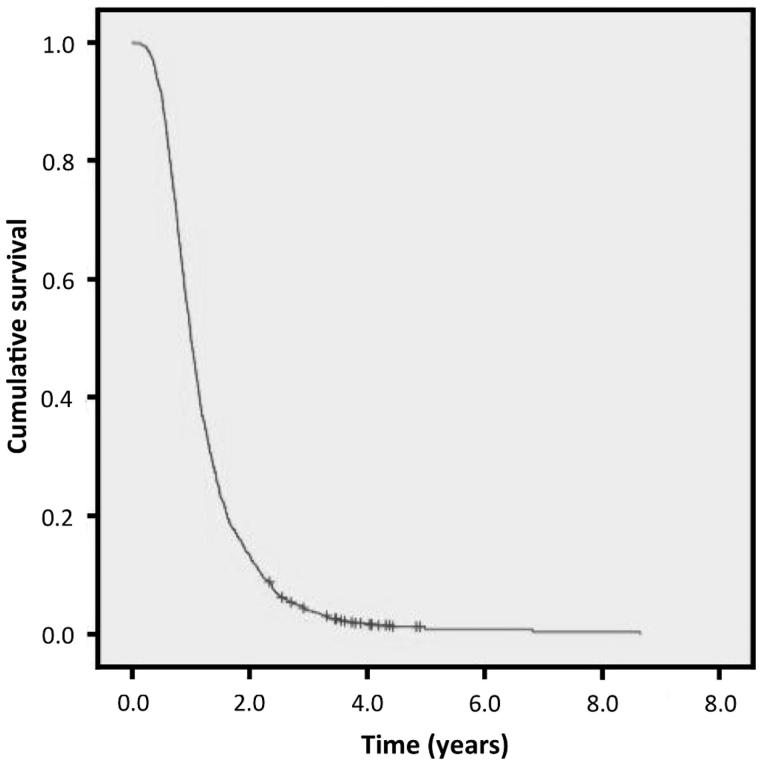
Kaplan–Meier survival curve for the whole population from which the study groups were derived.

The short and long survivors were of the same ancestry as the general UK ALS population as shown by principal components analysis (Supplementary Figure 1). There was no cryptic relatedness (pi-hat <0.05 in all pairwise comparisons). Short survivors lived at most 1.5 years from symptom onset, and long survivors at least six years. Demographic details for the study groups are shown in Table I. The Kaplan–Meier survival curve for the population from which the study groups were derived is shown in Figure 1. The median survival for this population is 2.76 years.

There were 16 rare variants passing quality control with MAF ≤0.05. Using burden testing as implemented in SKAT, we did not find association with survival of rare variants in *KIFAP3* (*p* = 1.0; six variants) or *EPHA4* (*p* = 0.49; two variants). SNVs in *UNC13A* were associated with survival (*p* = 6.57 × 10^−3^; eight variants) passing the Bonferroni corrected significance threshold of 0.017 (0.05/3).

Extending the analysis to include all variants with a frequency cut-off of MAF ≤0.125 did not change the results.

**Figure 2.**
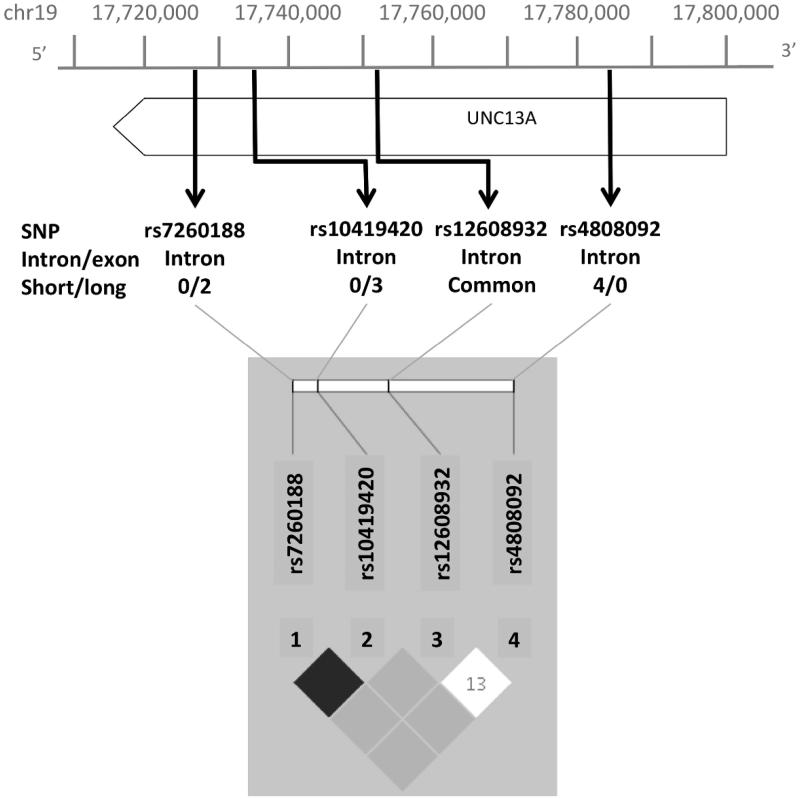
Visual representation of location of rare and common SNVs within UNC13A and associated LD plot. Intron/exon status and number of individuals in long/short survival groups given for rare SNVs.

Analysis of the rare variation within *UNC13A* driving the association, revealed three intronic SNVs, rs7260188:G > T, rs10419420:G > A and rs4808092:G > A (Figure 2). Minor alleles at SNVs rs7260188 and rs10419420 occurred only in the long survival group, in two and three individuals, respectively, and were in linkage disequilibrium (*r^2^* = 0.66) (Figure 2, Table II). SNV rs4808092:G > A was carried by four individuals in the short survival group only (Table IV).

**Table I.** Demographic features of the long and short survival groups (UK cohort).

	Long survival group	Short survival group
Number	25	25
Male:Female	10: 15	8: 17
Mean age of onset (y)	53.7	60.2
Proportion alive	0.6	0.04
Median survival (KM method) (y)	9.16	1.04

**Table II.** Linkage disequilibrium values for SNPs in *UNC13A* associated with extremes of survival in a UK population.

		D′	R^2^
rs7260188	rs10419420	1	0.66
rs7260188	rs12608932	1	0
rs7260188	rs4808092	1	0.001
rs10419420	rs12608932	1	0.016
rs10419420	rs4808092	1	0.001
rs12608932	rs4808092	0.139	0.001

Because a possible explanation for the association is linkage disequilibrium with the common variant rs12608932, A > C known to be associated with ALS survival (4,6), we examined the haplotypic and correlation structure of the rare and common variants together (Figure 2, Table II) and compared frequencies with results from the 1000 Genomes Project (Table III). We also repeated the SKAT analysis of *UNC13A* using the presence of the common variant rs12608932, A > C as a covariate which did not affect the results (*p* = 0.01). The rs7260188 SNV is monomorphic in 1000 Genomes EUR data. The findings support a difference in allele frequencies between ALS cases and 1000 Genomes controls. Similarly, there is a difference in allele frequency for the common SNV, rs12608932 with the C allele present in 55% of ALS chromosomes and 35% of 1000 Genomes EUR chromosomes, consistent with existing association studies of this SNV.

**Table III.** SNV minor allele frequency (MAF) for UK study population and 1000 Genomes Project.

	Minor allele frequency (MAF)	
SNV	UK study population	1000 Genomes Project
rs7260188	0.020	0.0
rs10419420	0.031	0.005
rs12608932	0.450	0.349
rs4808092	0.040	0.046

**Table IV.** Genotype count data for variants of interest in UK discovery cohort.

		Frequency in UK discovery cohort	
SNV	Genotypes	Long survival group (*n* = 25)	Short survival group (*n* = 25)
rs7260188	G G	23	25
	G t	2	0
	tt	0	0
rs10419420	G G	21	25
	G a	3	0
	aa	0	0
rs12608932	A A	3	3
	A c	18	15
	cc	4	7
rs4808092	G G	25	21
	G a	0	4
	aa	0	0

To replicate the findings, we studied a dataset from the Netherlands that had been selected, processed and sequenced in an almost identical way. There was no significant difference in allele frequencies between the long and short survivors, and SKAT analysis of rare variation in *UNC13A* revealed no significant difference between the long and short survival groups (*p* = 0.132; 271 variants).

**Figure 3.**
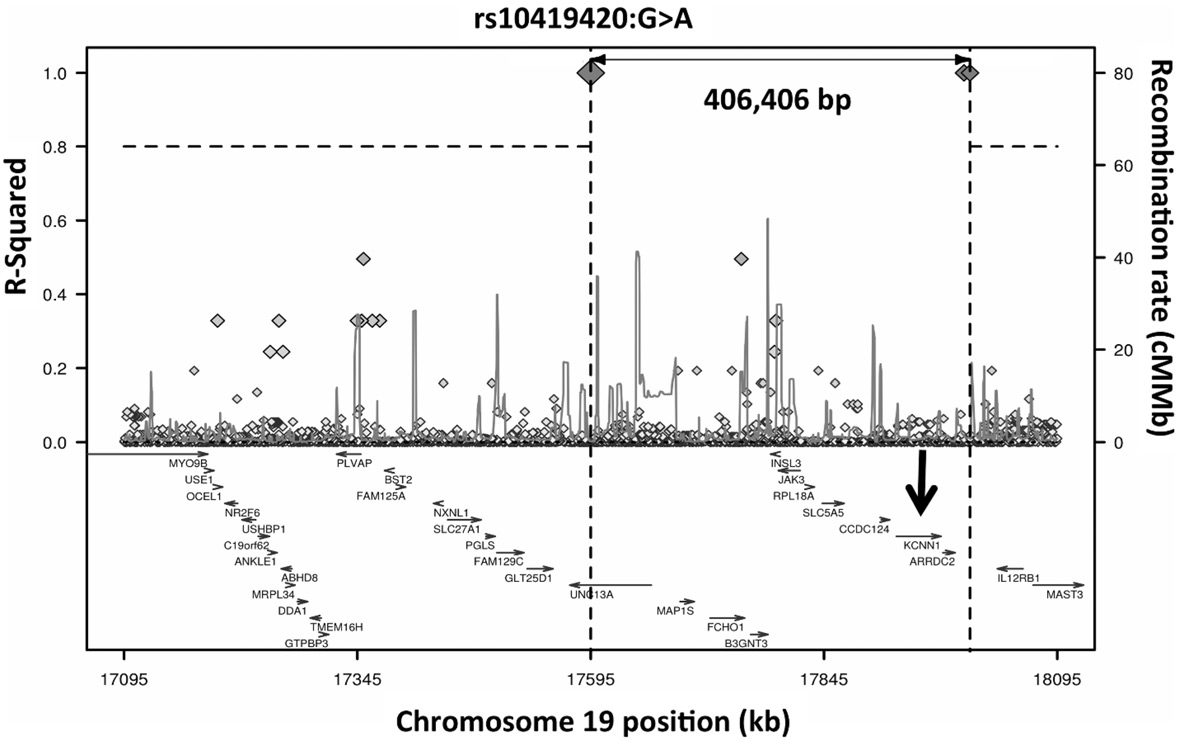
Long-range LD plot of UNC13A SNV rs10419420. The position of the KCNN1 gene, for which the common rs12608932 SNV in UNC13A acts as an eQTL, is shown with an arrow. Generated using SNP Annotation and Proxy Search (www.broadinstitute.org/mpg/snap/ldplot.php).

Surprisingly, SNV rs10419420 shows strong long range linkage disequilibrium in 1000 Genomes CEU data with rs77902754, >400Kb distant (*r^2^* = 0.99, Figure 3). This finding was not replicated in the Dutch dataset.

## Discussion

We have shown that rare variation in the *UNC13A* gene may modify survival in ALS in a UK sample set, but this is not replicated in a second sample set from the Netherlands. The association is independent from that of common variation at rs12608932, which is associated with ALS risk and survival (4,6,18). We have not found rare SNVs modifying ALS risk in the *EPHA4* or *KIFAP3* genes.

The rare variants identified in *UNC13A* in the UK sample were intronic and exclusively found in one or other extreme of survival. SNV rs10419420 variation was only seen in the long survivors, and rs4808092 variation only seen in the short survivors. The allele frequencies of the UK sample and population estimates from the 1000 Genomes EUR samples [www.1000genomes.org] are given in Table III. Seven of 50 individuals (14%) in this study carried one of the survival modifying variants.

There are a few possible explanations for the lack of replication of the finding. First, it is possible that the UK results represent a false-positive association. Secondly, rare variants are more likely to be population-specific than common variants, and it is therefore more likely that rare variation within a gene modifying survival varies by population, accounting for the differences identified between the populations sampled in this study. Of the three variants identified in the UK samples, one was monomorphic in the Dutch samples, and the other two had a minor allele frequency >5% and were not therefore rare.

Overall, population-specific effects are likely to represent a significant challenge affecting whole genome sequencing studies focusing on rare variation because the large population samples that may be required to replicate findings within different geographical cohorts will be extremely challenging to collect in relatively rare diseases. This is further compounded by the unknown significance of intronic variation and other ‘non-functional’ variation identified with whole-genome sequencing designs.

The common rs12608932 SNV in *UNC13A* has been identified as both an ALS risk (19,20) and survival (6,18) variant. This study adds to the body of evidence supporting *UNC13A* variants as important in modifying ALS survival and suggests that the pathological effect may not be confined to a function of rs12608932 or an associated variant.

The mechanistic role of *UNC13A* in ALS survival remains unclear, although it is known to play a role in neurotransmission. The mouse homolog of the *UNC13A* protein, Munc13, has been shown to play a role in neurotransmission with a central role in synaptic-vesicle priming (21). Munc13 deficiency has been shown to influence glucose homeostasis resulting in abnormal glucose tolerance (22). A number of studies have identified abnormal glucose metabolism in patients with ALS and a primary metabolic abnormality in ALS has been proposed (23,24). A study of a C. elegans model of ALS has shown that unc-13, the C. elegans homolog of *UNC13A*, plays a role in motor neuron degeneration through interaction with TDP-43, a key pathological protein in ALS (25,26). Thus, at least three potential mechanisms link *UNC13A* with motor neuron degeneration, and all could either increase ALS risk or affect the rate of neurodegeneration and, therefore, survival.

The rarer SNV associated with long survival, rs7260188 and the more common SNP rs10419420, are in strong linkage disequilibrium. SNV rs7260188 is monomorphic in 1000 Genomes EUR samples, and is only found in two of the three patients with the rs10419420 SNV; we have therefore focused analysis on rs10419420.

The linkage disequilibrium pattern of the three main variants, rs10419420 found in the long survivors, the common SNV rs12608932, and rs4808092, found in the short survivors is complex. The r^2^ relationships are all close to zero, both in this study and in public data. This is expected since a common variant will often have low r^2^ with a rare variant, because the allele frequencies are different. For the rare variants, the low r^2^ arises because the minor allele at rs10419420 lies on the common allele at rs4808092. The genotypes at any one locus are therefore independent of those at the others. Similarly, in both this study and the public data, the D’ and haplotype patterns show that for the rare SNVs, each rare allele lies on the common allele at the other marker (Table III) and there is no evidence for the fourth haplotype (both rare alleles). However, when the common SNV is included, the rs10419420 SNV, associated with long survival, is only seen on the common allele of the common SNV, while the rs4808092 SNV, associated with short survival, is seen on either haplotype. This means that there is a haplotype seen in the long survivors, AAG (2.5%), that is not seen at all in the 1000 Genomes data.

The SNV associated with long survival, rs10419420, is in very strong long-range LD with SNV rs77902754 (Figure 2, *r^2^* = 0.99). The rs77902754 SNV lies in a regulatory region comprising a transcription binding site, transcribed mRNAs and an H3K27Ac mark, and is very close to the KCNN1 gene, a calcium activated potassium channel which contributes to neuronal excitability. This proximity is important because the common rs12608932 SNV in *UNC13A* has been proposed to act as an expression quantitative trait locus (eQTL) for KCNN1 (27,28). Thus there are two independent signals relating variation in the *UNC13A* gene to the region around KCNN1, and although SKAT analysis does not directly identify an association between rare variants in KCNN1 and survival (*p* = 0.579), it remains possible that the signal seen in *UNC13A* is a proxy for biological effects conferred by KCNN1 or a different protein.

A weakness of this study is the small sample size limiting the statistical power of the study. We have attempted to mitigate this by selecting samples on the basis of extreme phenotype, which is known to increase power (12). We have also restricted the study only to candidate survival genes despite having whole-genome sequence data, thus reducing the multiple testing burden. We have used a SKAT burden test, which improves power to detect association for rare variant studies because it allows detection of protective and deleterious alleles at the same locus. A strength of the study is the detailed sequencing of the candidate genes, allowing detection of all rare and common variation.

Our study highlights the potential for extreme phenotype design in the search for survival genes and rare variants associated with ALS.

## Supplementary Material

Sup1_accepted.tif

## Notes

**Supplementary material available online**
