# Supplementary figures and images for: Rare genetic variation in UNC13A may modify survival in amyotrophic lateral sclerosis
Source: Amyotroph Lateral Scler Frontotemporal Degener. 2016 Sep 1;17(7-8):593–9. doi: 10.1080/21678421.2016.1213852 (PMC5125285; doi:10.1080/21678421.2016.1213852)

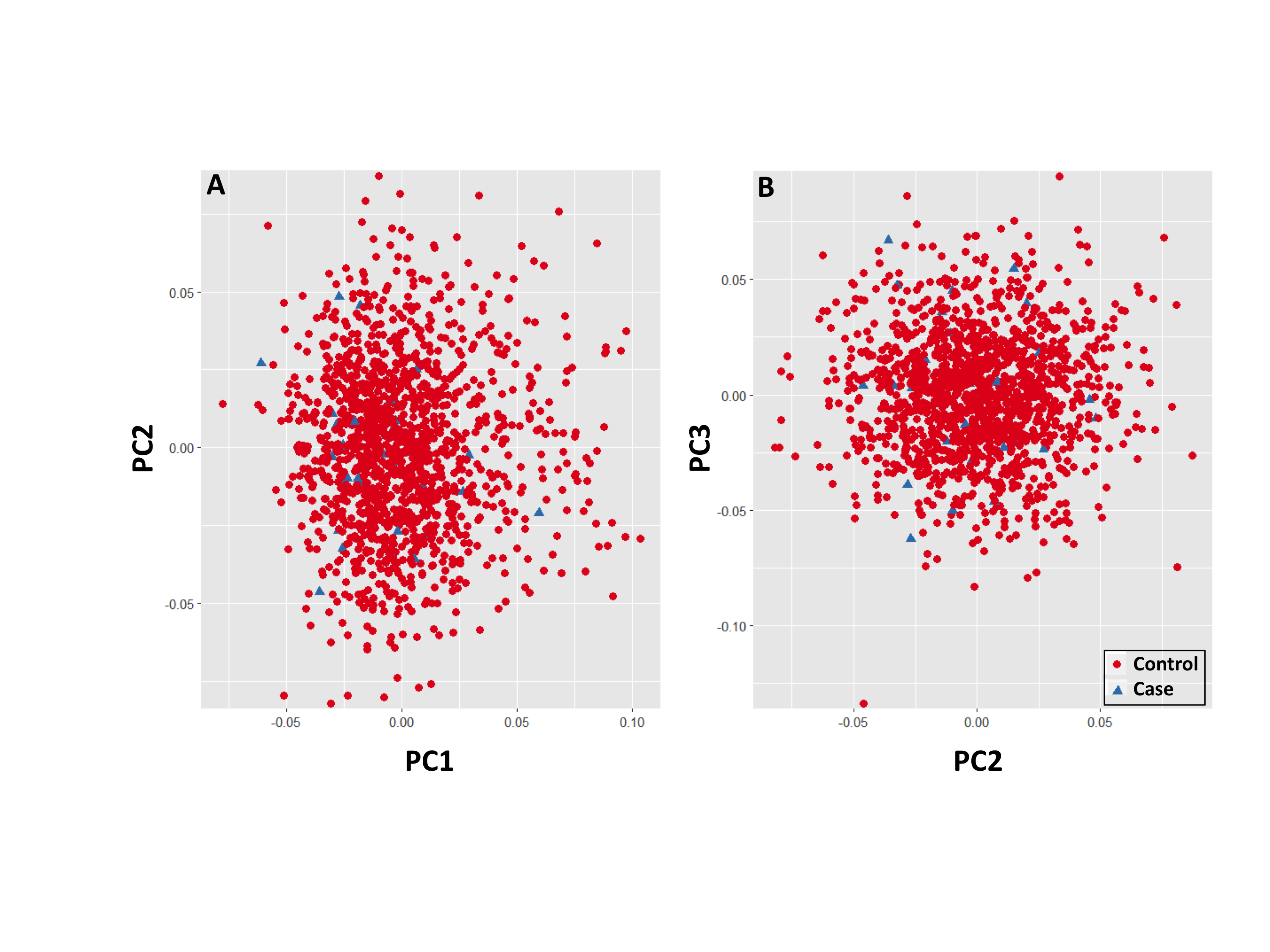

Supplement: Sup1_accepted.tif [file iafd_a_1213852_sm3211.tif]
